# Diabetes, Biochemical Markers of Bone Turnover, Diabetes Control, and Bone

**DOI:** 10.3389/fendo.2013.00021

**Published:** 2013-03-08

**Authors:** Jakob Starup-Linde

**Affiliations:** ^1^Faculty of Health, Aalborg UniversityAalborg, Denmark; ^2^Department of Endocrinology and Internal Medicine, Aarhus University Hospital THGAarhus, Denmark

**Keywords:** diabetes mellitus, bone, bone turnover, markers of bone turnover, biochemical markers, glycemic control

## Abstract

Diabetes mellitus is known to have late complications including micro vascular and macro vascular disease. This review focuses on another possible area of complication regarding diabetes; bone. Diabetes may affect bone via bone structure, bone density, and biochemical markers of bone turnover. The aim of the present review is to examine *in vivo* from humans on biochemical markers of bone turnover in diabetics compared to non-diabetics. Furthermore, the effect of glycemic control on bone markers and the similarities and differences of type 1- and type 2-diabetics regarding bone markers will be evaluated. A systematic literature search was conducted using PubMed, Embase, Cinahl, and SveMed+ with the search terms: “Diabetes mellitus,” “Diabetes mellitus type 1,” “Insulin dependent diabetes mellitus,” “Diabetes mellitus type 2,” “Non-insulin dependent diabetes mellitus,” “Bone,” “Bone and Bones,” “Bone diseases,” “Bone turnover,” “Hemoglobin A Glycosylated,” and “HbA1C.” After removing duplicates from this search 1,188 records were screened by title and abstract and 75 records were assessed by full text for inclusion in the review. In the end 43 records were chosen. Bone formation and resorption markers are investigated as well as bone regulating systems. T1D is found to have lower osteocalcin and CTX, while osteocalcin and tartrate-resistant acid are found to be lower in T2D, and sclerostin is increased and collagen turnover markers altered. Other bone turnover markers do not seem to be altered in T1D or T2D. A major problem is the lack of histomorphometric studies in humans linking changes in turnover markers to actual changes in bone turnover and further research is needed to strengthen this link.

## Introduction

Diabetes mellitus is a common disease in most parts of the world (WHO, 2012; Wikipedia, 2012). Well known late complications of diabetes are micro vascular disease including nephropathy, retinopathy, neuropathy, and macro vascular disease such as acute coronary syndrome, claudicatio intermittens, and stroke (American Diabetes Association, 2012). However, the bone turnover and thus the skeletal integrity may also be affected by diabetes, and diabetic bone disease can represent a hitherto overlooked complication of diabetes. A meta-analysis reported lower bone mineral density (BMD) *z*-score in T1D, but higher *z*-score in T2D compared to controls (Vestergaard, 2007). Despite the higher BMD, patients with T2D have more fractures than non-diabetic controls (Vestergaard, 2007). Patients with T1D also have more hip fractures than can be explained by the decreased BMD (Vestergaard, 2007). The increased fracture risk is supported by a larger Danish study, where diabetics without late complications had a relative risk of any fracture of 1.21 (1.07–1.36) for T1D and of 1.13 (1.06–1.22) for T2D (Vestergaard et al., 2009). This may point at a weakening of bone biomechanical competence beyond what can be measured by BMD. This disruption of biomechanical competence may be brought about by alterations in bone turnover and non-calcium bone matrix (such as collagen), as BMD mainly reflects calcium content in the bone.

### Bone turnover in general

**Figure 1:**
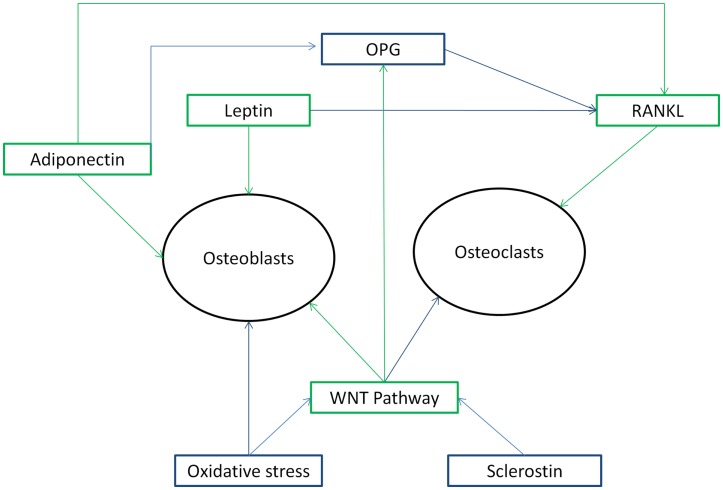
**Overview of different systems regulating bone metabolism in its relationship with each other and effects on osteoblasts and osteoclasts as mentioned in the Section “Introduction” above**. Figure 1 shows that the systems regulating bone metabolism are in a complex relationship to each other. Green arrows indicate a stimulating action, while blue arrows indicate a inhibiting action.

Bone turnover is a dual relationship between the process of bone formation by osteoblasts (creation of new bone) and the process of bone resorption by osteoclasts (removal of old bone) (Delmas, 1991; Garnero, 2009). Bone markers are subdivided into bone formation and bone resorption markers. Bone formation markers consist of osteocalcin (OC), bone-specific alkaline phosphatase (BAP), alkaline phosphatase (AP), osteoprotegerin (OPG), procollagen type 1 amino terminal propeptide (P1NP), and procollagen type 1 carboxyl terminal propeptide (P1CP) (Delmas, 1991; Garnero, 2009), while resorptive markers consist of N-terminal cross-linked telopeptide of type-I collagen (NTX), C-terminal cross-linked telopeptide of type-I collagen (CTX), tartrate-resistant acid phosphatase (TRAP), RANKL (Receptor Activator of Nuclear factor Kappa beta Ligand), pyridinoline (PYR), deoxypyridinoline (DPD), hydroxyproline (HP), and sclerostin (Scl) (Delmas, 1991; Manolagas and Almeida, 2007; Garnero, 2009). Scl, OPG, and RANKL are not markers in strict sense, but are included in this section, since they are related to bone turnover (see below). These markers represent products secreted by cells such as OC and Scl and enzymes (alkaline and acid phosphatase), collagen cleavage products as examples of the organic matrix, and calcium itself. Figure 1 gives an overview of some of the different systems (described below) regulating bone turnover.

### Biochemical markers secreted by bone cells

(a) Osteocalcin is a valid marker of bone turnover both when formation and resorption are uncoupled as well as when formation and resorption are coupled (Delmas, 1991). However, OC fluctuates with food intake making it susceptible to error and it may be questioned if the serum marker fully reflects what is going on in the bone in diabetics. OC becomes γ-carboxylated at three glutamine terminals so it can interact with hydroxyapatite, when fewer than three terminals are γ-carboxylated it is undercarboxylated OC (ucOC) (Motyl et al., 2010). Besides being a marker of bone formation, OC and UcOC are also associated with beta-cell function and insulin sensitivity and thus control of plasma glucose levels (Lee et al., 2007; Ferron et al., 2008; Confavreux et al., 2009). UcOC is suggested to stimulate the secretion of adiponectin from adipocytes (Motyl et al., 2010; Ng, 2011). In mice, OC injections prevent T2D and improve glucose control (Ferron et al., 2012). Furthermore, a human study concluded that OC is associated with improved glucose tolerance and insulin secretion (Hwang et al., 2012). Glycemic control has been linked to increased OC (Motyl et al., 2010; Bao et al., 2011), although the mechanisms are unclear. (b) Both AP and BAP are bone formation markers measured in serum. While BAP is specific for bone, AP consists of different iso-enzymes like the hepatic AP (Delmas, 1991). (c) TRAP is an enzyme in different subtypes including an osteoclast specific enzyme (TRAP-5b) (Garnero, 2009). TRAP-5b is a marker that reflects the number and activity of osteoclasts and shows little variation regarding time of day and food intake (Garnero, 2009). (d) Scl is a Wnt-pathway antagonist produced in the osteocytes (Gennari et al., 2012). The Wnt-pathway promotes osteoblastogenesis, increases OPG expression, and decreases osteoclastogenesis and bone resorption (Manolagas and Almeida, 2007).

### Biochemical markers of collagen

P1NP and P1CP are secreted to the blood after their cleavage from collagen (Delmas, 1991). Both P1NP and CTX fluctuate rapidly with glucose intake (Clowes et al., 2003), and may thus be less precise. The rapid fluctuations with glucose intake make it questionable if these markers truly reflect what is going on in the bone during hyperglycemia. PYR is found in collagen of bone, but also in other tissues, while DPD is only at significant amounts in bone collagen (Delmas, 1991). PYR and DPD are mature collagen crosslinks released by collagen breakdown and is thus measurable markers (Calvo et al., 1996). Some of these crosslinks are released in peptide-bound forms (CTX, NTX), which are shown useful in bone assessment (Calvo et al., 1996; Achemlal et al., 2005). Since DPD is a limited marker, it seems more specific and sensitive regarding bone resorption than PYR (Miazgowski and Czekalski, 1998). HP is found in collagens of different tissues and after its release, during collagen breakdown, most of it is reabsorbed in the kidneys and oxidized in the liver. HP thus does not reflect the total collagen catabolism (Delmas, 1991).

### Calcium

Serum (s-) calcium is tightly regulated by the calcitropic hormones (see below) and thus often is a poor marker of bone turnover. Urinary (u-) calcium may be used as a marker of bone resorption, but is rather variable with calcium intake and thus requires standardized sampling. For these reasons it is rarely used as a marker of bone turnover in clinical practice. In diabetics, hyperglycemia may be associated with increased loss of calcium in the urine (Raskin et al., 1978; McNair et al., 1979).

### Calcitropic hormones

The calcium, PTH, and vitamin D system is tightly related to bone metabolism. PTH is secreted from the parathyroid glands and is a regulator of 1,25OHD production, calcium absorption, and bone calcium release. PTH inhibits Scl production in humans (Garcia-Martin et al., 2012a), thereby influencing bone formation by multiple pathways. Vitamin D acts by stimulating the intestinal absorption of calcium and is a regulator of the calcium homeostasis. Vitamin D stimulates the synthesis of OC (Inaba et al., 1999). Calcitonin affects osteoclasts by inhibiting bone resorption and affects the kidney by enhancing urinary calcium excretion (Pondel, 2000).

### The OPG/RANKL system

The OPG/RANKL system is a part of the bone turnover metabolism. RANKL is the agonist in regulating important aspects of osteoclasts like differentiation, fusion, survival, activation, and apoptosis (Horowitz et al., 2001). RANKL acts by activating a specific receptor (RANK – Receptor Activator of Nuclear factor Kappa beta) on the osteoclasts and promoting bone resorption, making RANKL a marker of bone resorption (Horowitz et al., 2001). In contrast, OPG is the antagonist to RANKL (Galluzzi et al., 2005). Another inhibitor of RANKL seems to be hyperglycemia, which induces low bone turnover and suppresses osteoclastogenesis (Wittrant et al., 2008). Again the question is whether the serum markers truly reflect what is going on in the bone as no biopsy studies are available.

### Bone–fat interaction

Fat tissue is suggested to be an actor in both diabetes and bone metabolism. T2D patients are often overweight and present with issues related to this (American Diabetes Association, 2012).

(a) Adiponectin is secreted by fat tissue, and a receptor for adiponectin on osteoblasts has been identified (Berner et al., 2004; Kanazawa et al., 2007). Adiponectin is suggested to stimulate proliferation, differentiation, and mineralization of osteoblasts (Berner et al., 2004; Luo et al., 2005; Kanazawa et al., 2007). It is proposed that adiponectin plays a role in energy metabolism by increasing insulin sensitivity, reducing endogenous glucose production, and decreasing serum glucose levels (Berg et al., 2001; Combs et al., 2001). Adiponectin affects the RANKL/OPG system by stimulating RANKL and inhibiting OPG production in osteoblasts, thereby promoting osteoclast formation and bone resorption (Luo et al., 2006). (b) Another fat tissue marker, leptin, is found to suppress osteoclast and bone resorption *in vitro* by inhibiting RANKL and may increase bone mass (Holloway et al., 2002; Reid, 2002). Leptin also increases proliferation and differentiation of osteoblasts (Reid, 2002).

### The IGF system and advanced glycation end products

Another aspect of bone turnover is the effect of insulin and insulin like products. Insulin like growth factor-1 (IGF-1) is an important anabolic regulator of the bone, which mainly is produced in the liver but other tissues as well like in the osteoblasts (Verhaeghe et al., 1992; Johansson et al., 1996; Yakar et al., 2002). In T2D, IGF levels may be increased (Frystyk et al., 1999). In addition insulin is believed to promote osteoblastogenesis and increase bone formation (Reid et al., 1993; Reid, 2002). In contrast, oxidative stress and the production of advanced glycation end products (AGE) are associated with inhibition of osteoblast differentiation and to osteoblast apoptosis, thus leading to reduced osteoblast function (Alikhani et al., 2007; Hamada et al., 2009). Oxidative stress also antagonizes the before mentioned Wnt-pathway and therefore inhibits osteoblast activity (Manolagas and Almeida, 2007).

### Aim

The aim of the present review is to:

(a) Examine *in vivo* human evidence on the connection between diabetes mellitus and bone markers compared to non-diabetics and collate the different markers with each other.
(b) Evaluate the effect of glycemic control in diabetics on bone markers and assess the similarities and differences between type 1- and type 2-diabetics.

## Methods

To perform this review a literature search was conducted in association with a research librarian. The databases, PubMed, Embase, Cinahl, SveMed+, Cochrane library, and Bibliotek.dk were screened using the search terms: “Diabetes mellitus,” “Diabetes mellitus type 1,” “Insulin dependent diabetes (IDD) mellitus,” “Diabetes mellitus type 2,” “Non-insulin dependent diabetes (NIDD) mellitus,” “Bone,” “Bone and Bones,” “Bone diseases,” “Bone turnover,” “Hemoglobin A Glycosylated,” and “HbA1C.” In total 1,188 records were retrieved from the literature search. Duplicates were removed and records screened by title and abstract. The records were screened so they are of a cross-sectional, retrospective, case-control, or prospective design. The eligibility criteria to the studies are; that they shall examine bone turnover markers in relationship to diabetics with or without a control group. Studies assessing the effect of different medications were excluded. By these criteria 1,113 records were removed. The remaining 75 records were assessed in full text for inclusion in the review by the same eligibility criteria as above. In the end 43 records were included in this review. Of the 43 records; 32 were of a cross-sectional design, and 11 were of a prospective design.

Some of the records did not subdivide diabetics in T1D and in T2D, but rather subdivided as IDD and NIDD. The IDD and NIDD subdivision is mainly used in studies of older date (all before year 2000). In the following, IDD will be treated as T1D and NIDD will be treated as T2D. However insulin treated T2D may be included in IDD, while NIDD cannot be T1D (which is an IDD type). This may be a setback, still only four studies used IDD as diabetes subdivision (see Tables 1–4), which makes the usage of T1D and IDD as one, a minor issue to the review. Furthermore when glycemic control and 7 years of insulin therapy are mentioned it means a significant decrease in HbA1c or a decrease in FPG. The study using FPG do not conclude whether the drop is significant (Gregorio et al., 1994).

**Table 1:** **Calcitropic system markers**.

Marker	Study type	Reference	Diabetes type	*
s-Calcium	Cross-sectional	Dobnig et al. (2006)	T2D	↑
		Levy et al. (1986)	T2D	↑
		Rasul et al. (2012a)	T2D women vs. men	↑
		Pedrazzoni et al. (1989)	IDD men	↑
			NIDD women	↑
			NIDD men	↑
		Achemlal et al. (2005)	T2D men	→
		Gregorio et al. (1994)	NIDD PMC	→
			NIDD GMC	→
		Hampson et al. (1998)	T1D PM	→
			T2D PM	→
		Shu et al. (2012)	T2D PM	→
		Cutrim et al. (2007)	T2D GMC	→
			T2D PMC	→
		Zhou et al. (2010)	T2D PM (BMI ≥25)	→
			T2D PM (BMI <25)	→
		Garcia-Martin et al. (2012b)	T2D	→
		Oz et al. (2006)	T2D	→
		Rasul et al. (2012b)	T2D men PNP	→
			T2D women PNP	→
		Neumann et al. (2011)	T1D women	→
			T1D men	→
		Abd El Dayem et al. (2011)	T1D	→
		Hamed et al. (2011)	T1D	↓
	Longitudinal	Hamilton et al. (2012)	T2D	↑
			T1D	→
	Glycemic control	Campos Pastor et al. (2000)	T1D	↑
		Capoglu et al. (2008)	T2D	→
u-Calcium	Cross-sectional	Achemlal et al. (2005)	T2D men	→
		Gregorio et al. (1994)	NIDD PMC	→
			NIDD GMC	→
		Oz et al. (2006)	T2D	→
	Longitudinal	Inaba et al. (1999) (mg/mg)	T2D DS	↑
PTH	Cross-sectional	Hamed et al. (2011)	T1D	↑
		Gennari et al. (2012)	T1D	↑
		Gregorio et al. (1994)	NIDD PMC	↑
		Galluzzi et al. (2005)	T1D	→
		Neumann et al. (2011)	T1D women	→
		Hampson et al. (1998)	T1D PM	→
		Gennari et al. (2012)	T2D	→
		Hampson et al. (1998)	T2D PM	→
		Achemlal et al. (2005)	T2D men	→
		Oz et al. (2006)	T2D	→
		Shu et al. (2012)	T2D PM	→
		Cutrim et al. (2007)	T2D GMC	→
			T2D PMC	→
		Zhou et al. (2010)	T2D PM (BMI ≥25)	→
				→
		Gregorio et al. (1994)	NIDD GMC	→
		Rasul et al. (2012a)	T2D men vs. women	→
		Rasul et al. (2012b)	T2D men PNP	→
			T2D women PNP	→
		Gerdhem et al. (2005)	Diabetic women	→
		Neumann et al. (2011)	T1D men	↓
		Pedrazzoni et al. (1989)	IDD women	↓
			IDD men	↓
			NIDD women	↓
			NIDD men	↓
		Garcia-Martin et al. (2012b)	T2D	↓
		Dobnig et al. (2006)	T2D NH	↓
		Reyes-Garcia et al. (2013)	T2D	↓
		Okuno et al. (2013)	Diabetics HD	↓
	Longitudinal	Hamilton et al. (2012)	T1D men	→
			T1D women	→
			T2D men	→
			T2D women	→
		Inaba et al. (1999)	T2D DS	↓
	Glycemic control	Campos Pastor et al. (2000)	T1D	↑
		Capoglu et al. (2008)	T2D	→
		Rosato et al. (1998)	NIDD men	→
			NIDD women	→
		Gregorio et al. (1994)	NIDD PMC	↓
25OHD	Cross-sectional	Galluzzi et al. (2005)	T1D	→
		Garcia-Martin et al. (2012b)	T2D	→
		Gregorio et al. (1994)	NIDD GMC	→
			NIDD PMC	→
		Hampson et al. (1998)	T2D PM	→
		Shu et al. (2012)	T2D PM	→
		Cutrim et al. (2007)	T2D GMC	→
			T2D PMC	→
		Dobnig et al. (2006)	T2D NH	→
		Reyes-Garcia et al. (2013)	T2D	→
		Rasul et al. (2012a)	T2D men vs. women	→
		Rasul et al. (2012b) (nM/l)	T2D men PNP	→
			T2D women PNP	→
		Gerdhem et al. (2005)	Diabetic women	→
		Gennari et al. (2012)	T1D	↓
		Hampson et al. (1998)	T1D PM	↓
		Hamed et al. (2011)	T1D	↓
		Gennari et al. (2012)	T2D	↓
	Longitudinal	Hamilton et al. (2012)	T1D	→
			T2D	↓
	Glycemic control	Capoglu et al. (2008)	T2D	→
		Rosato et al. (1998)	NIDD men	→
			NIDD women	→
1,25OHD	Cross-sectional	Gregorio et al. (1994)	NIDD GMC	→
			NIDD PMC	→
		Shu et al. (2012)	T2D PM	→
		Rasul et al. (2012b)	T2D men PNP	→
			T2D women PNP	→
		Rasul et al. (2012a)	T2D men vs. women	→
Calcitonin	Cross-sectional	Pedrazzoni et al. (1989)	IDD women	↑
			IDD men	↑
			NIDD women	↑
			NIDD men	↑
		Gregorio et al. (1994)	NIDD GMC	→
			NIDD PMC	→
		Zhou et al. (2010)	T2D PM (BMI ≥25)	→
			T2D PM (BMI <25)	→

Glycemic control (during the longitudinal study); *, statistically significantly different; ↑, significantly higher in diabetics; ↓, significantly lower in diabetics; →, without significance; GMC, good metabolic control; PMC, poor metabolic control; PM, postmenopausal; NH, nursing home; age ≥70; HD, hemodialysis; PNP, polyneuropathy.

## Data on Markers

**Figure 2:**
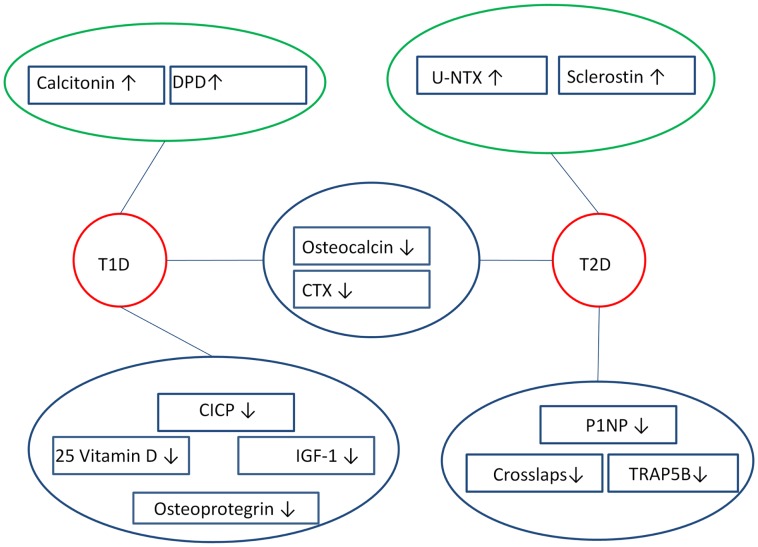
**Overview of bone markers that are likely to differ in diabetics compared to controls**. Both T1D and T2D appear to have altered bone resorption markers and bone formation markers, where bone formation markers (OC, P1NP, CICP) are lowered, while bone resorption markers (DPD, u-NTX, CTX, TRAP) are either lowered or raised. This reveals a dissociation in the bone markers among diabetics, as bone markers that do not differ are left out of the figure. ↑ Indicates raised marker, ↓ indicates lowered marker, blue circle surrounds markers that appear to be lowered, green circle surrounds markers that seem to be raised, red circle surrounds diabetics.

Table 1 shows details on the calcitropic system in diabetics, Table 2 shows details on bone formation markers in diabetics, Table 3 shows details on bone resorption markers in diabetics, and Table 4 shows details on other bone markers in diabetics. An overview of the markers that seem to differ in diabetics is shown in Figure 2.

**Table 2:** **Bone formation markers**.

Marker	Study type	Reference	Diabetes type	*
AP	Cross-sectional	Cutrim et al. (2007)	T2D PMC	↑
		Pedrazzoni et al. (1989)	NIDD men	↑
		Pedrazzoni et al. (1989)	IDD men	↑
		Cutrim et al. (2007)	T2D GMC	→
		Oz et al. (2006)	T2D	→
		Shu et al. (2012)	T2D PM	→
		Zhou et al. (2010)	T2D PM (BMI ≥25)	→
		Zhou et al. (2010)	T2D PM (BMI <25)	→
		Hamed et al. (2011)	T1D	→
		Pedrazzoni et al. (1989)	IDD women	→
		Pedrazzoni et al. (1989)	NIDD women	↓
	Longitudinal	Miazgowski and Czekalski (1998)	IDD	↑
		Hamilton et al. (2012)	T1D men	↑
			T1D women	↑
			T2D men	→
			T2D women	→
	Glycemic control	Campos Pastor et al. (2000)	T1D	→
BAP	Cross-sectional	Kanazawa et al. (2011b)	T2D PM vs. men	↑
		Rasul et al. (2012b)	T2D women PNP	↑
		Gennari et al. (2012)	T1D	→
		Garcia-Martin et al. (2012b)	T2D	→
		Shu et al. (2012)	T2D PM	→
		Reyes-Garcia et al. (2013)	T2D	→
		Gerdhem et al. (2005)	Diabetic women	→
		Okuno et al. (2013)	Diabetic HD	→
		Rasul et al. (2012a)	T2D men vs. women	→
		Gennari et al. (2012)	T2D	↓
		Oz et al. (2006)	T2D	↓
		Rasul et al. (2012b)	T2D men PNP	↓
	Longitudinal	Miazgowski et al. (2012)	T2D PM	→
		Inaba et al. (1999)	T2D	→
	Glycemic control	Kanazawa et al. (2011a)	T2D	↑
		Campos Pastor et al. (2000)	T1D	→
		Capoglu et al. (2008)	T2D	↓
		Kanazawa et al. (2009b)	T2D	↓
OC	Cross-sectional	Aboelasrar et al. (2010)	T1D PrP vs. Pb	↑
		Rasul et al. (2012b)	T2D men PNP	↑
		Gregorio et al. (1994)	NIDD PMC	↑
		Gennari et al. (2012)	T1D	→
		Pedrazzoni et al. (1989)	IDD women	→
		Leon et al. (1989)	T1D	→
		Gennari et al. (2012)	T2D	→
		Garcia-Martin et al. (2012b)	T2D	→
		Gregorio et al. (1994)	NIDD PMC	→
		Cutrim et al. (2007)	T2D GMC	→
		Reyes-Garcia et al. (2013)	T2D	→
		Rasul et al. (2012a)	T2D men vs. women	→
		Rasul et al. (2012b)	T2D women PNP	→
		Neumann et al. (2011)	T1D men	↓
			T1D women	↓
		Abd El Dayem et al. (2011)	T1D	↓
		Aboelasrar et al. (2010)	T1D	↓
		Lappin et al. (2009)	T1D LH	↓
			T1D HH	↓
		Danielson et al. (2009)	T1D	↓
		Brandao et al. (2007)	T1D girls vs. boys	↓
		Pedrazzoni et al. (1989)	IDD men	↓
		Christensen and Svendsen (1999)	IDD PrM vs. PM	↓
			NIDD PrM vs. PM	↓
		Pedrazzoni et al. (1989)	NIDD women	↓
			NIDD men	↓
		Achemlal et al. (2005)	T2D men	↓
		Oz et al. (2006)	T2D	↓
		Dobnig et al. (2006)	T2D NH	↓
		Shu et al. (2012)	T2D PM	↓
		Cutrim et al. (2007)	T2D PMC	↓
		Zhou et al. (2010)	T2D PM (BMI ≥25)	↓
			T2D PM (BMI <25)	↓
		Gregorio et al. (1994)	NIDD GMC	↓
		Kanazawa et al. (2011b)	T2D men vs. PM	↓
		Gerdhem et al. (2005)	Diabetic women	↓
	Longitudinal	Miazgowski and Czekalski (1998)	IDD	↑
		Hamilton et al. (2012)	T1D men	→
			T1D women	→
			T2D men	→
			T2D women	→
		Mastrandrea et al. (2008)	T1D <20	→
		Mastrandrea et al. (2008)	T1D ≥20	↓
	Glycemic control	Kanazawa et al. (2009b)	T2D	↑
		Rosato et al. (1998)	NIDD men	↑
			NIDD women	↑
		Kanazawa et al. (2011a)	T2D	→
		Campos Pastor et al. (2000)	T1D	→
		Capoglu et al. (2008)	T2D	↓
		Gregorio et al. (1994)	NIDD PMC	↓
ucOC	Cross-sectional	Rosato et al. (1998)	NIDD men	↑
			NIDD women	↑
		Okuno et al. (2013)	Diabetic HD	↓
	Glycemic control	Kanazawa et al. (2009b)	T2D	→
P1NP	Cross-sectional	Rasul et al. (2012b)	T2D men PNP	↑
		Rasul et al. (2012a)	T2D men vs. women	→
		Rasul et al. (2012b)	T2D women PNP	→
		Shu et al. (2012)	T2D PM	↓
PICP	Cross-sectional	Cutrim et al. (2007)	T2D GMC	→
			T2D PMC	→
CICP	Cross-sectional	Hampson et al. (1998)	T1D PM	→
			T2D PM	→
		Oz et al. (2006)	T2D	→
		Lappin et al. (2009)	T1D LH	→
			T1D HH	→
		Abd El Dayem et al. (2011)	T1D	↓

Glycemic control (during the longitudinal study); *, statistically significantly different; ↑, significantly higher in diabetics; ↓, significantly lower in diabetics; →, without significance; GMC, good metabolic control; PMC, poor metabolic control; PM, postmenopausal; NH, nursing home; age ≥70; HD, hemodialysis; PNP, polyneuropathy; PrM, premenopausal; LH, low HbA1c <8.5; HH, high HbA1c >8.5; ≥20, 20 years old or more; <20, younger than 20 years; Pb, pubertal; PrP, prepubertal.

**Table 3:** **Bone resorption markers**.

Marker	Study type	Reference	Diabetes type	*
TRAP	Glycemic control	Campos Pastor et al. (2000)	T1D	↓
TRAP-5b	Cross-sectional	Okuno et al. (2013)	Diabetic HD	→
		Reyes-Garcia et al. (2013)	T2D	↓
		Garcia-Martin et al. (2012b)	T2D	↓
CTX	Cross-sectional	Rasul et al. (2012b)	T2D men PNP	↑
		Neumann et al. (2011)	T1D women	→
		Achemlal et al. (2005)	T2D men	→
		Shu et al. (2012)	T2D PM	→
		Rasul et al. (2012a)	T2D men vs. women	→
		Rasul et al. (2012b)	T2D women PNP	→
		Gennari et al. (2012)	T1D	↓
		Gennari et al. (2012)	T2D	↓
		Neumann et al. (2011)	T1D men	↓
		Garcia-Martin et al. (2012b)	T2D	↓
		Oz et al. (2006)	T2D	↓
		Dobnig et al. (2006)	T2D NH	↓
		Reyes-Garcia et al. (2013)	T2D	↓
		Gerdhem et al. (2005)	Diabetic women	↓
		Brandao et al. (2007)	T1D girls vs. boys	↓
	Longitudinal	Hamilton et al. (2012)	T2D women	↑
			T2D men	→
			T1D men	→
			T1D women	→
s-NTX	Cross-sectional	Shu et al. (2012)	T2D PM	→
		Danielson et al. (2009)	T1D	→
u-NTX	Cross-sectional	Zhou et al. (2010)	T2D PM (BMI ≥25)	↑
			T2D PM (BMI <25)	↑
		Kanazawa et al. (2011b)	T2D PM vs. men	↑
	Longitudinal	Mastrandrea et al. (2008)	T1D <20	→
			T1D ≥20	→
	Glycemic control	Kanazawa et al. (2011a)	T2D	→
		Kanazawa et al. (2009b)	T2D	→
		Capoglu et al. (2008)	T2D	↓
DPD	Cross-sectional	Abd El Dayem et al. (2011)	T1D	↑
		Hampson et al. (1998)	T2D PM	↑
		Hampson et al. (1998)	T1D PM	→
		Valerio et al. (2002)	T1D	→
		Cutrim et al. (2007)	T2D GMC	→
			T2D PMC	→
		Oz et al. (2006)	T2D	→
		Gerdhem et al. (2005)	Diabetic women	↓
	Longitudinal	Miazgowski and Czekalski (1998)	IDD	→
		Inaba et al. (1999)	T2D	→
		Miazgowski et al. (2012)	T2D PM	→
	Glycemic control	Rosato et al. (1998)	NIDD men	→
			NIDD women	→
		Capoglu et al. (2008)	T2D	↓
PYR	Longitudinal	Miazgowski and Czekalski (1998)	IDD	→
		Inaba et al. (1999)	T2D	→
		Rosato et al. (1998)	NIDD men	↑
			NIDD women	↑
Crosslaps	Cross-sectional	Christensen and Svendsen (1999)	IDD PM vs. PrM	↑
			NIDD PM vs. PrM	↑
HP	Cross-sectional	Gregorio et al. (1994)	NIDD PMC	↑
			NIDD GMC	→

Glycemic control (during the longitudinal study); *, statistically significantly different; ↑, significantly higher in diabetics; ↓, significantly lower in diabetics; →, without significance; GMC, good metabolic control; PMC, poor metabolic control; PM, postmenopausal; NH, nursing home; age ≥70; HD, hemodialysis; PNP, polyneuropathy; PrM, premenopausal; ≥20, 20 years old or more; <20, younger than 20 years.

**Table 4:** **Other markers**.

Marker	Study type	Reference	Diabetes type	*
OPG	Cross-sectional	Galluzzi et al. (2005)	T1D	↑
		Lappin et al. (2009)	T1D LH	↑
			T1D HH	↑
		Abd El Dayem et al. (2011)	T1D	↓
RANKL	Cross-sectional	Lappin et al. (2009)	T1D LH	→
			T1D HH	→
Scl	Cross-sectional	Garcia-Martin et al. (2012b)	T2D	↑
		Gennari et al. (2012)	T2D	↑
Adiponectin	Cross-sectional	Kanazawa et al. (2011b)	T2D PM vs. men	↑
	Longitudinal	Miazgowski et al. (2012)	T2D PM	↑
	Glycemic control	Kanazawa et al. (2009b)	T2D	→
IGF-1	Cross-sectional	Cutrim et al. (2007)	T2D GMC	→
			T2D PMC	→
		Hamed et al. (2011)	T1D	↓
	Longitudinal	Mastrandrea et al. (2008)	T1D <20	↑
			T1D ≥20	→
	Glycemic control	Rosato et al. (1998)	NIDD men	↑
			NIDD women	↑

Glycemic control (during the longitudinal study); *, statistically significantly different; ↑, significantly higher in diabetics; ↓, significantly lower in diabetics; →, without significance; GMC, good metabolic control; PMC, poor metabolic control; PM, postmenopausal; LH, low HbA1c <8.5; HH, high HbA1c >8.5.

### Calcium

For data on s-calcium and u-calcium, see Table 1. In summary, s-calcium and u-calcium seem not to differ between either T1D or T2D and controls. S-calcium is higher in T2D women than men, with evidence from one study that this may be caused by their postmenopausal state (Rasul et al., 2012a), while another was not informative on this (Pedrazzoni et al., 1989). S-calcium may show a small but significant increase in T2D (2.1 vs. 2.4 mmol/l) (Hamilton et al., 2012) over time and poor glycemic control may result in a fall in u-calcium.

### Parathyroid hormone

For data on s-PTH, see Table 1. It is unlikely that renal dysfunction has affected the results, since one study adjusted by creatinine clearance (Dobnig et al., 2006), while all others, expect one (Gerdhem et al., 2005), excluded participants with renal impairment. In summary, s-PTH is likely to be variable in T1D and T2D, since it has been reported to be unchanged, higher, and lower. In T2D the absence of a difference is most likely as it was found by the majority of studies. S-PTH seems not to correlate to BMD in T1D or T2D nor is it likely to differ over time in T1D and T2D, although Vitamin D stimulation decreases s-PTH. Glycemic control is, in T1D, likely to result in a rather large increase in s-PTH, while glycemic control in T2D most likely does not change s-PTH.

### Serum 1,25 vitamin D and 25 vitamin D

For data regarding 1,25 vitamin D and 25 vitamin D, see Table 1. To summarize S-25OHD is likely to be lower in T1D than controls, while both s-25OHD and s-1,25OHD are most likely not to differ between T2D and controls, since the majority of studies reported no difference. S-25OHD may decrease over time in T2D, but not in T1D. The lower s-25OHD levels in T2D may be due to an increased mean age of these individuals (Hamilton et al., 2012). Furthermore glycemic control seems not change s-25OHD in T2D.

### Calcitonin

For data on calcitonin, see Table 1. In summary, calcitonin seems higher in T1D (*P* > 0.05) than controls, although it is in normal value range, and not to differ in T2D in comparison to controls. However, the number of studies is limited.

### Alkaline phosphatase

For data on s-AP, see Table 2. To sum up s-AP seems not to differ in T1D and T2D in comparison to controls, since the largest studies showed no difference (Oz et al., 2006; Zhou et al., 2010; Hamed et al., 2011; Shu et al., 2012). Over time s-AP may increase in T1D, which can be caused by hepatic involvement (Hamilton et al., 2012), since other bone markers (s-OC, s-CTX, u-PYR, u-DPD) do not differ. S-AP is also reported not to differ in T1D and T2D over time, nor to change by glycemic control in T1D.

### Bone-specific alkaline phosphatase

For data on s-BAP, see Table 2. In summary, s-BAP is most likely not to differ in either T1D or T2D in comparison to controls. S-BAP seems lower in T2D males than T2D females, which may reflect the postmenopausal state in the females (Kanazawa et al., 2011b). S-BAP may not correlate to HbA1c or change over time in T2D, nor is it likely to change by glycemic control in both T1D and T2D.

### Osteocalcin

For data on s-OC, see Table 2. In summary, s-OC is likely to be up to four times lower in young T1D than controls (12.2 vs. 49.4 ng/ml) (Abd El Dayem et al., 2011) and somewhat lower in older T1D than controls. A negative relationship to pubertal development is probable in T1D, whereas s-OC may normalize in adulthood. S-OC is likely not to correlate to BMD in T1D, but to have a positive relationship to s-CTX and a negative relationship to HbA1c. In T2D s-OC is most likely to be somewhat lower than among controls, as the studies reporting a lower s-OC includes larger populations. Also s-OC is probably negatively associated with HbA1c in T2D. Regarding the longitudinal studies; s-OC is most likely not to change in T1D and T2D over time, while glycemic control neither seem to change s-OC in T1D. However, in T2D, glycemic control may either not change, decrease, or increase s-OC, where the studies finding a decrease were the ones including the longest period of time and therefore supporting a decrease. Overall, changes in s-OC are likely to relate to changes in HbA1c.

### Undercarboxylated osteocalcin

For data on s-ucOC, see Table 2. To sum up, s-ucOC seems twice as low in diabetics on hemodialysis (14.4 vs. 31.5 ng/ml) (Okuno et al., 2013) than matched controls and almost half the level in T2D males compared females, which may be explained by the postmenopausal state in the females. S-ucOC appears positively related to bone turnover markers and negatively to HbA1c, while glycemic control does not to change ucOC.

### Procollagen type 1 amino terminal propeptide, procollagen type 1 C propeptide, and collagen type 1 C propeptide

For data on s-P1NP, s-PICP, and s-CICP, see Table 2. To summarize, s-P1NP is likely to be somewhat lower in T2D than controls (see Table 2). On the other hand, neither s-PICP nor s-CICP, are likely to differ regarding T2D, while s-CICP may be somewhat lower in T1D supported by a larger study population (see Table 2). S-CICP is also probable to be negatively correlated to BMD in T1D.

### Tartrate-resistant acid phosphatase

For data on s-TRAP, see Table 3. S-TRAP-5b is likely to be much lower in T2D than controls. Also s-TRAP seems not to correlate to HbA1c; however it seems to undergo a somewhat large decrease during glycemic control in T1D.

### Carboxy-terminal telopeptide of type 1 collagen

For data on s-CTX, see Table 3. In summary, s-CTX is very likely to be up to twice as low in T2D and T1D, when both are compared to controls, since both are reported with convincing significance (*P* < 0.001). Over time s-CTX seems to increase twofold in comparison to the baseline value (0.26 vs. 0.13 ng/ml) in T2D females, but not in T2D males or T1D, which could be caused by the probable postmenopausal state due to higher mean age in these T2D females (70 at follow up), while T1D females had a lower mean age (53 at follow up) (Hamilton et al., 2012).

### N-telopeptide

For data on N-telopeptide, see Table 3. To summarize, s-NTX is unlikely to differ in either T2D or T1D, but seems to have a negative association with HbA1c in T1D. However, few studies are available.

### Urinary N-terminal cross-linked telopeptide of type-I collagen

For data on u-NTX, see Table 3. To sum up, u-NTX is likely to be higher in T2D than controls and be almost twice as low in T2D men as women (see Table 3), which may relate to the fact that these women are postmenopausal (Kanazawa et al., 2011b). U-NTX is in T2D likely to correlate negatively to BMD, but not to correlate to HbA1c. Over time, u-NTX does not seem to change in T1D. During glycemic control through 12 months T2D may decrease in u-NTX (Capoglu et al., 2008), while shorter periods do not seem to affect u-NTX.

### Deoxypyridinoline, pyridinoline

For data on u-DPD and u-PYR, see Table 3. In summary, u-DPD may be twice the value in young T1D as controls (Abd El Dayem et al., 2011), while it seems to normalize in adulthood. In T2D, u-DPD is likely not to differ, although unspecified diabetics seem to have lower u-DPD than controls. U-DPD seems not to correlate to BMD in T1D. Nor is u-DPD likely to change over time in T1D and T2D as well as u-PYR does not change in T1D. Furthermore, during glycemic control u-PYR seems to increase in T2D, while u-DPD, on the other hand, is likely to decrease.

### Hydroxyproline and crosslaps

For data on u-HP and u-crosslaps, see Table 3. In summary, u-crosslaps is likely to be lower in T2D than controls. U-HP seems to be positively associated with HbA1c (Gregorio et al., 1994).

### Osteoprotegerin and RANKL

For data on OPG and RANKL, see Table 4. In summary s-RANKL is likely not to differ in T1D, while s-OPG may be lower in T1D, since this is supported by a larger study.

### Sclerostin

For data on s-Scl, see Table 4. Also, s-Scl tended to correlate positively to HbA1c levels in T2D patients, however the correlation was not statistically significant (Garcia-Martin et al., 2012b; Gennari et al., 2012). In relation to BMD, s-Scl is in T2D reported to positively relate to *T*-score in lumbar spine, femoral neck, and total hip (*P* < 0.05) (Garcia-Martin et al., 2012b) and s-Scl levels are lower in osteoporotic- than non-osteoporotic-T2D (*P* = 0.048) (Garcia-Martin et al., 2012b). In summary, s-Scl is likely to be higher in T2D than both T1D and controls. S-Scl tends to be positively related to HbA1c and is likely to be positively related to BMD in T2D.

### Adiponectin and leptin

For data on adiponectin, see Table 4. In summary, s-adiponectin is likely to be higher in T2D women than T2D men, which may be due to postmenopausal state among the women studied (Kanazawa et al., 2009a). It is uncertain whether s-adiponectin is related to bone turnover markers, while s-leptin seems to have a negative relationship to u-NTX. Moreover glycemic control seems not to change s-adiponectin in T2D.

### IGF-1

For data on s-IGF, see Table 4. To summarize, s-IGF-1 is likely to be somewhat lower in T1D and not to differ in T2D. Over time, s-IGF-1 seems to decrease in younger T1D, while it did not differ in older T1D. This may be caused by a depletion of the insulin function in the young T1D at follow up. Also, during glycemic control IGF-1 seems to increase in T2D.

## Discussion

In general a major issue is the few histomorphometric studies in humans (Leite Duarte and da Silva, 1996; Armas et al., 2012), and the absence of studies on the association between biochemical markers of bone turnover in blood and actual changes in bone tissue. Several markers, especially OC, CTX, and P1NP may also vary with blood glucose or glucose intake, making them perhaps less markers of bone turnover in diabetics and more markers of alterations in glucose metabolism. Another issue is kidney function, which may influence the measurement of several biochemical markers of bone turnover and also influence histomorphometry of the bone (Andress et al., 1987).

### Type 1 diabetics vs. non-diabetics

Neither s-calcium nor u-calcium seems to be specific markers of bone in T1D or differ in comparison to controls (Hampson et al., 1998; Brandao et al., 2007; Abd El Dayem et al., 2011; Neumann et al., 2011), although young T1D might have a lower s-calcium (Hamed et al., 2011). Hence may the bone deficiency, by lower BMD and increased fracture risk (Vestergaard, 2007; Vestergaard et al., 2009) in T1D, be formed in childhood, leaving the bones fragile in adulthood, while calcium levels normalize.

Regarding bone turnover markers secreted by bone cells; s-AP, s-BAP, and s-Scl seem not to differ in T1D compared to controls (Leon et al., 1989; Munoz-Torres et al., 1996; Oz et al., 2006; Hamed et al., 2011; Gennari et al., 2012), and s-TRAP seems in normal value range (Munoz-Torres et al., 1996). S-OC appear to be lower in T1D and may decrease during the pubertal growth (Brandao et al., 2007; Aboelasrar et al., 2010; Abd El Dayem et al., 2011); so those diagnosed before adulthood are bone growth impaired and the bone affection may continue in adulthood (Danielson et al., 2009; Lappin et al., 2009). The lack of an association of s-OC with BMD (Munoz-Torres et al., 1996; Brandao et al., 2007; Danielson et al., 2009) and the positive relationship to s-CTX (Brandao et al., 2007; Abd El Dayem et al., 2011) suggest that formation and resorption are coupled processes in T1D, and may not affect BMD. The negative associations of s-OC to HbA1c (Danielson et al., 2009; Aboelasrar et al., 2010; Abd El Dayem et al., 2011) suggest that bone formation is impaired by high levels of blood glucose; accordingly T1D with poor glycemic control has more fragile bones than T1D with good glycemic control.

When looking at bone turnover markers of collagen; s-CTX and s-CICP are likely to be lower in T1D (Abd El Dayem et al., 2011; Neumann et al., 2011; Gennari et al., 2012) and u-DPD may be higher in young T1D (Abd El Dayem et al., 2011), while u-crosslaps and s-NTX do not differ (Christensen and Svendsen, 1999; Danielson et al., 2009). Seemingly s-CICP is raised, while bone resorption markers may not differ, be raised or be lowered, thus making it difficult to conclude definitively. The absence of a correlation of HbA1c and S-CTX (Lappin et al., 2009), and a negative correlation to s-NTX (Danielson et al., 2009) suggest that high HbA1c may affect bone resorption negatively. In young T1D, s-CTX seems negatively related to pubertal development (Brandao et al., 2007), suggesting that during pubertal development bone resorption is impaired in T1D. Taken together with the corresponding finding regarding s-OC, bone turnover seems to be reduced during pubertal development in T1D. The RANKL/OPG system has only been investigated in T1D, where bone resorption appears to be affected by a reduced OPG. Even so, s-CTX does not relate to s-OPG, but a positive relationship is apparent with s-RANKL and the RANKL/OPG ratio (Lappin et al., 2009). Seemingly, s-CTX is a marker of activity in the RANKL system and might be the end product of the process. Increased blood glucose seems not to be the mechanism that suppress s-OPG, since s-OPG is found positively correlated to HbA1c (Galluzzi et al., 2005; Lappin et al., 2009), indicating that increasing levels of blood glucose inhibit bone resorption.

### Type 2 diabetics vs. non-diabetics

Neither s-calcium nor u-calcium differed between T2D and controls (Gregorio et al., 1994; Hampson et al., 1998; Achemlal et al., 2005; Oz et al., 2006; Cutrim et al., 2007; Zhou et al., 2010; Garcia-Martin et al., 2012b; Shu et al., 2012). S-calcium appears not to correlate to HbA1c or BMD (Levy et al., 1986; Hampson et al., 1998), thus making it a poor marker of bone- and glycemic-status in T2D. The PTH-vitamin D axis; S-PTH, 1,25OHD, s-25OHD, and calcitonin are most likely not to be affected in T2D (Pedrazzoni et al., 1989; Gregorio et al., 1994; Hampson et al., 1998; Achemlal et al., 2005; Dobnig et al., 2006; Oz et al., 2006; Cutrim et al., 2007; Zhou et al., 2010; Garcia-Martin et al., 2012b; Gennari et al., 2012; Shu et al., 2012; Reyes-Garcia et al., 2013). The positive relationship between s-PTH and the resorptive markers s-TRAP-5b and s-CTX (Reyes-Garcia et al., 2013) suggests that PTH induce bone resorption in T2D.

Concerning bone turnover markers secreted by bone cells; s-AP and s-BAP seem not to differ in T2D (Oz et al., 2006; Zhou et al., 2010; Garcia-Martin et al., 2012b; Shu et al., 2012; Reyes-Garcia et al., 2013). S-OC and s-TRAP are likely to be decreased in T2D (Rosato et al., 1998; Achemlal et al., 2005; Dobnig et al., 2006; Oz et al., 2006; Zhou et al., 2010; Garcia-Martin et al., 2012b; Shu et al., 2012; Reyes-Garcia et al., 2013) and s-Scl, which impair bone formation (Manolagas and Almeida, 2007; Gennari et al., 2012), seems increased, suggesting that T2D is in a state of low bone turnover. S-OC seems and s-Scl tends to correlate negatively to HbA1c in T2D (Dobnig et al., 2006; Kanazawa et al., 2009a; Garcia-Martin et al., 2012b; Gennari et al., 2012), thus suggesting that the low bone turnover and bone deficiency in T2D may be caused by elevated glycemic levels. S-BAP have a negative relationship with IGF-1 (Kanazawa et al., 2009a, 2011b), while s-OC is in positive relationship with IGF-1 (Kanazawa et al., 2009a), proposing that IGF-1 is a marker of bone formation in T2D specific to OC. S-BAP, s-TRAP-5b, and s-CTX correlated negatively to s-Scl (Garcia-Martin et al., 2012b; Gennari et al., 2012), thus Scl correlates to decreased bone turnover, while it in contrast correlated to increased BMD (Garcia-Martin et al., 2012b). The increase in BMD is inconsistent with the antagonizing effect on the Wnt-pathway (Manolagas and Almeida, 2007). When looking at bone turnover markers of collagen; u-NTX is higher (Zhou et al., 2010) and s-NTX, s-PICP, s-CICP, and u-DPD seem not to differ (Hampson et al., 1998; Oz et al., 2006; Cutrim et al., 2007; Shu et al., 2012), while u-crosslaps, s-CTX, and s-P1NP are lower (Christensen and Svendsen, 1999; Dobnig et al., 2006; Oz et al., 2006; Garcia-Martin et al., 2012b; Gennari et al., 2012; Shu et al., 2012; Reyes-Garcia et al., 2013), suggesting that bone turnover is changed in T2D. The differences in bone resorption markers may reflect different points of progress in T2D bone affection or the fact that markers may be sensitive or insensitive in T2D. The markers may also be affected by the heterogeneity of the T2D state. Also, S-CTX is negatively related to and P1NP tends to negatively relate to HbA1c (Achemlal et al., 2005; Dobnig et al., 2006; Shu et al., 2012), while u-HP is positively related to HbA1c (Gregorio et al., 1994) and u-NTX does not relate to HbA1c (Kanazawa et al., 2009a). Apparently elevated levels of blood glucose suppress bone formation, and decrease or increase markers of bone resorption in T2D, which is consistent with the findings concerning s-OC and s-Scl. Therefore blood glucose may have taken part in the previous mentioned fluctuating pattern of the resorption markers. The negative relationship between S-CTX, u-NTX, and BMD (Reyes-Garcia et al., 2013), suggest these markers as informants on BMD and that extensive bone resorption cause low BMD.

Concerning fat tissue hormones; adiponectin and leptin may have a positive effect on bone status by suppressing resorptive markers, increasing bone formation markers and BMD (Tamura et al., 2007; Kanazawa et al., 2011b). However, this is uncertain and further studies are needed.

### Type 1 vs. type 2 diabetics

The lack of a difference in bone turnover markers indicate that T1D and T2D (Hampson et al., 1998; Gennari et al., 2012) are not different regarding the effect on bone markers, although Scl levels are higher in T2D (Gennari et al., 2012), proposing that bones are affected through an antagonizing effect on the WNT-pathway in T2D, but not in T1D. However the full selection of bone markers is not represented.

### How severity of disease affects markers

Severity of disease is characterized by diabetics with polyneuropathy or diabetics in hemodialysis. S-P1NP is reported higher in T2D with polyneuropathy than regular T2D otherwise no markers differ (OC and s-BAP) (Rasul et al., 2012b). In diabetics in hemodialysis s-ucOC and s-PTH are lower, while s-TRAP-5b and s-BAP do not differ in comparison to equally ill controls (Okuno et al., 2013). More severe diabetes is likely to affect bone markers by raising P1NP and lowering s-ucOC, this may be caused by the severe disease itself.

### Duration of diabetes and the effect on bone

S-OC in T1D (Abd El Dayem et al., 2011) and S-TRAP-5b in T2D (Reyes-Garcia et al., 2013), are negatively correlated to the duration of diabetes. This suggests that long term diabetes lead to suppressed bone turnover and thus potentially fragile bones.

### The effect of time on markers in type 1 diabetics

The markers s-OC, s-CTX, u-PYR, u-DPD are likely not to differ over time (Miazgowski and Czekalski, 1998; Mastrandrea et al., 2008; Hamilton et al., 2012), suggesting that bone turnover does not change over time in T1D and support the hypothesis that bone turnover is lowered during puberty, since none of the available longitudinal studies have investigated a young T1D population.

### The effect of time on markers in type 2 diabetics

The resorption marker s-CTX increase over 5 years, while no other bone turnover marker seems to change (Miazgowski and Czekalski, 1998; Hamilton et al., 2012), suggesting either no change in bone turnover over time, thus the s-CTX increase may be seen by chance, or that s-CTX is a specific marker in T2D, where others are not. An increase in s-25OHD may affect this; even so PTH did not change (Miazgowski and Czekalski, 1998; Hamilton et al., 2012). The s-adiponectin increase before it normalized back to baseline level in newly diagnosed in the study by Miazgowski et al. (2012) suggest, together with a negative relationship to BMD, that a quick rise and following normalization in s-adiponectin may lower BMD in T2D. On the other hand, changes in femoral neck BMD seems to relate positively to baseline log [adiponectin] (Kanazawa et al., 2010), which does not accord to the above. Over time HbA1c does not seem to be related to u-DPD, s-BAP (Kanazawa et al., 2010; Miazgowski et al., 2012), suggesting that HbA1c does not affect bone turnover markers.

### The changes by glycemic control

Seven years of intensive insulin therapy decrease s-TRAP, while bone formation markers did not change in T1D (Campos Pastor et al., 2000), indicating that glycemic control alters bone resorption, while it has no effect on bone formation.

The intervention of glycemic control is, in T2D, executed by regular diabetes control, diet, exercise, or medical treatment. The glycemic control seems not to change s-BAP, ucOC, s-PTH, s-25OHD, and s-adiponectin, while u-PYR and s-IGF-1 seem to increase, and u-DPD, u-HP, u-calcium, and u-NTX seem to decrease (Gregorio et al., 1994; Rosato et al., 1998; Capoglu et al., 2008; Kanazawa et al., 2009b). S-OC may not change, increase, or decrease during glycemic control (Gregorio et al., 1994; Capoglu et al., 2008; Kanazawa et al., 2009b, 2011a), where a short period of glycemic control increase s-OC, intermediate periods decrease s-OC, and the longest available period of glycemic control (2 years) increase s-OC (Rosato et al., 1998). The previously proposed positive effect of glycemic control on OC may be present, however the link between OC and glycemic control seems ambiguous due to the different reports. In general; bone resorption may decrease during glycemic control, although u-PYR increase, and bone formation may not change. PYR is not as good a bone marker as DPD, since it is not specific for bone tissue, so to say it is most likely that bone resorption is lowered in T2D, and the PYR increase could be due to collagen breakdown at non-bone sites, suggesting glycemic control to decrease bone resorption.

### Cohorts

The data on biochemical markers was collected from several records as mentioned in the Section “Methods.” In general the studies used small cohorts consisting of around 50 diabetics and a similar control group. The studies on T2D have larger cohorts than studies on T1D, whereas the largest T1D cohort is of 128 participants (Neumann et al., 2011), while the largest diabetic cohort by far is found in Zhou et al. (2010) where 890 postmenopausal T2D were examined. Another aspect is that the cohorts were very heterogeneous in age and postmenopausal status among women. Most studies on T2D included have a mean age around 60 and if they included women, they were postmenopausal, although other studies have older or younger populations. In opposition the studies examining T1D primarily looked at younger populations and a larger fraction of the studies assessed bone status in children and/or adolescents. The studies have different means by assessing diabetes in their participant; some use criteria by WHO or American Diabetes Association, while others retrieve the patients from hospitals and outpatient clinics and a study does not mention how they got their diabetic participants (Okuno et al., 2013). However all these methods, except the last mentioned, seem reliable and therefore the results seem to consider diabetics. Another problem in comparing the results of the studies is the difference in diabetes duration. T1D cohorts have been reported with mean diabetes duration varying from 2.67 (Hamed et al., 2011) to 18.5 years (Gennari et al., 2012). For T2D the variation in time since diagnosis spans from a diagnosis made within the last year (Miazgowski et al., 2012) to 14.3 years (Tamura et al., 2007). This is an issue, since diabetes duration seems to affect bone turnover markers negatively and therefore makes it hard to compare the studies with different disease durations. Regarding the longitudinal studies; their period of follow up differs from 1 month (Kanazawa et al., 2009b) to 7 years (Campos Pastor et al., 2000), which may question the comparability of the results. Many confounders are also likely to influence the results including co morbidities, diabetes medication, other medications, and baseline characteristics as BMI, smoking, and alcohol. Even though most studies exclude participants with bone metabolism related diseases and bone metabolism affecting treatment their exclusion criteria are not unanimous. Also T2D is in the available records medically treated very differently. Some receive only diet changes, while others receive oral anti diabetics and others again insulin or a mix of different treatment modalities. This is a subject of concern regarding the reliability of the comparability of the different results.

The small cohorts, the heterogeneity among the studies regarding age, menopausal state, diabetes duration, exclusion criteria, and diabetes treatment may affect the results on the bone turnover, whereas truly hidden bone affection may be hidden in the large number of confounders.

### Closing remarks

The paradox of increased Scl and yet increased BMD in T2D, may be partly explained by inflammation. Inflammation is a part of both the T1D and the T2D disease (Bending et al., 2012; Calle and Fernandez, 2012). This may affect the bone markers and Scl as in inflammatory diseases, which is linked to bone resorption and osteoporotic fractures (Lacativa and Farias, 2010). Hence the effect on certain bone markers, as concluded in this review, may be due to inflammation.

The differences in bone turnover markers may also relate to the fact, that some markers are specific in diabetics, while others are not. The specificity of markers could be due to diabetes *per se* or the results of diabetes by blood glucose alterations, insulin deficiency, and AGE. Elevated blood glucose may result in measurement errors regarding bone markers, which could explain these differences. In addition cohort differences may influence the findings and hide the true effect of diabetes on bone markers by confounders. In experimental rat models, where the human confounders are not present, bone markers as OC, PYR, and TRAP-5b are reported decreased in diabetic rats in comparison to controls (Herrero et al., 1998; Suzuki et al., 1998). Therefore supporting that bone markers and bone turnover are lowered in the diabetic state and the fluctuating pattern in humans may be due to other effects than diabetes *per se*. However the findings regarding rats may not be transferable to humans. In conclusion the alteration of bone turnover in T1D and T2D may be mediated through elevated blood glucose levels and long duration of diabetes.

### Perspectives

In general bone formation and resorption are tightly coupled, and formation markers and resorptive markers tend to change in a coordinated way. The dissociation seen in diabetes, where some markers decrease (both formation markers, such as OC in both T1D and T2D, and resorption markers such as CTX and TRAP in T2D), whereas the remainder do not (e.g., AP) could point to a very specific uncoupling effect on these of factors associated with the disruption in glucose metabolism in diabetes. Perhaps glucose alters the circulating levels without affecting bone turnover *per se*. This may be supported by the only histomorphometric study in humans with T1D, which showed no alteration in bone turnover (Armas et al., 2012). However, more research is needed, perhaps including modern PET scanning techniques using fluoride to elucidate bone turnover (Puri et al., 2012).

## Conflict of Interest Statement

The authors declare that the research was conducted in the absence of any commercial or financial relationships that could be construed as a potential conflict of interest.

## Abbreviations

1,25OHD, 1,25 dihydroxy vitamin D; 25OHD, 25 hydroxy vitamin D; AP, alkaline phosphatase; BAP, bone-specific alkaline phosphatase; BMD, bone mass density; BMI, body mass index; CICP, collagen type 1 C propeptide; CTX, C-terminal cross-linked telopeptide of type-I collagen; DPD, deoxypyridinoline; FPG, fasting plasma glucose; HbA1C, glycated hemoglobin; HP, hydroxyproline; IDD, insulin dependent diabetes; IGF-1, insulin like growth factor-1; NIDD, non-insulin dependent diabetes; NTX, N-terminal cross-linked telopeptide of type-I collagen; OC, osteocalcin; OPG, osteoprotegerin; P1CP, procollagen type 1 carboxyl terminal propeptide; P1NP, procollagen type 1 amino terminal propeptide; PTH, parathyroid hormone; PYR, pyridinoline; RANK, receptor activator of nuclear factor kappa beta; RANKL, receptor activator of nuclear factor kappa beta ligand; S-, serum; Scl, sclerostin; T1D, type 1 diabetes; T2D, type 2 diabetes; TRAP, tartrate-resistant acid phosphatase; U-, urine; ucOC, undercarboxylated osteocalcin.
